# Urbanization Increases Pathogen Pressure on Feral and Managed Honey Bees

**DOI:** 10.1371/journal.pone.0142031

**Published:** 2015-11-04

**Authors:** Elsa Youngsteadt, R. Holden Appler, Margarita M. López-Uribe, David R. Tarpy, Steven D. Frank

**Affiliations:** 1 Department of Entomology, North Carolina State University, Raleigh, North Carolina, United States of America; 2 W. M. Keck Center for Behavioral Biology, North Carolina State University, Raleigh, North Carolina, United States of America; Universidade de São Paulo, Faculdade de Filosofia Ciências e Letras de Ribeirão Preto, BRAZIL

## Abstract

Given the role of infectious disease in global pollinator decline, there is a need to understand factors that shape pathogen susceptibility and transmission in bees. Here we ask how urbanization affects the immune response and pathogen load of feral and managed colonies of honey bees (*Apis mellifera* Linnaeus), the predominant economically important pollinator worldwide. Using quantitative real-time PCR, we measured expression of 4 immune genes and relative abundance of 10 honey bee pathogens. We also measured worker survival in a laboratory bioassay. We found that pathogen pressure on honey bees increased with urbanization and management, and the probability of worker survival declined 3-fold along our urbanization gradient. The effect of management on pathogens appears to be mediated by immunity, with feral bees expressing immune genes at nearly twice the levels of managed bees following an immune challenge. The effect of urbanization, however, was not linked with immunity; instead, urbanization may favor viability and transmission of some disease agents. Feral colonies, with lower disease burdens and stronger immune responses, may illuminate ways to improve honey bee management. The previously unexamined effects of urbanization on honey-bee disease are concerning, suggesting that urban areas may favor problematic diseases of pollinators.

## Introduction

With the global growth of cities, urbanization increasingly shapes the emergence and trajectory of infectious disease [1, 2]. The effects of urbanization on human diseases—such as measles and malaria—are variable but well documented [2, 3]. With few exceptions, however, the outcomes for other host-pathogen systems remain poorly known [4, 5]. Urbanization is expected to alter disease transmission and susceptibility in humans and other organisms by altering host population density and behavior, as well as resource quality and distribution [3, 5]. Understanding these complex effects will be critical to protecting wildlife, domestic animals, and ecosystem services on a rapidly urbanizing planet.

Pollination is an ecosystem service threatened by disease. Wild and managed insect pollinators have declined in abundance over the past century, with pathogens at least partly to blame for these losses [6–9]. Honey bees (*Apis mellifera*) are the principal providers of commercial crop pollination and honey in urban and rural systems worldwide [10]. The emergence and global homogenization of several pathogens is contributing to ongoing losses of honey bee colonies in the U.S. and Europe, threatening food security and the economic stability of beekeeping [11–14]. Central among these emerging pathogens are the fungal parasite *Nosema ceranae* and the parasitic mite *Varroa destructor*, along with a suite of viruses vectored or activated by the mite. *Nosema ceranae* and *V*. *destructor* shifted from their native host, *Apis cerana*, to *A*. *mellifera* in the past 50 years, and were first detected in the U.S. in 1995 and 1987, respectively [15–17]. *Nosema ceranae* attacks the honey bee gut, causing energetic stress and reducing the life span of individual bees [18]. *Varroa*-associated viruses, such as deformed wing virus (DWV), have been present in honey bees indefinitely, but their new interaction with mites has elevated them to previously unobserved severity and prevalence [12, 16]. Individually and in combination, these diseases cause severe colony losses in beekeeping operations [18, 19], and infection with multiple disease agents is linked to the sudden losses observed in colony collapse disorder (e.g., [20]).

Feral honey bee populations—historically or recently escaped from management and living without human intervention—also crashed in the wake of *Varroa* introductions [11, 12]. A growing number of reports, however, document feral populations coexisting stably with *Varroa* or *N*. *ceranae* in the absence miticide treatments or other management [21–24]. Feral colonies capable of overwintering without beekeeper support may exhibit immune traits that enable them to combat or tolerate pathogens. Detecting such patterns and, ultimately, identifying their genetic or environmental mechanisms would be key steps toward sustainable pollination services.

Managed and feral colonies are regularly found in and around cities, and we expect urbanization to alter their disease ecology. This has not previously been tested, and the effects of urbanization on whole-colony performance are equivocal (e.g., see contrasting results in [25] and [26]). By concentrating bees on fragmented resource patches, cities should increase opportunities for horizontal disease transmission [5, 27]. Patchy urban habitats may also demand long, costly foraging flights that exact a trade-off with expensive insect immune functions and induce oxidative stress [28, 29]. Moreover, urban warming may alter the costs of colony thermoregulation [30] and favor warm-climate disease agents such as *N*. *ceranae* [31, 32]. Conversely, the unusually diverse pollen resources available in urban gardens may improve nutrition and support immunity [33–36], while pollutants such as heavy metals can enhance or suppress immunity [37, 38]. On balance, however, the effects of urbanization largely favor susceptibility and transmission of disease agents, and we expect urbanization to reduce immune response and increase intensity of pathogen infections. Further, we expect the effects of urbanization to be more intense in feral colonies than managed colonies, since the response of feral bees to their environment is unmitigated by management.

Here we test these predictions by examining the effects of urbanization, management, and their interaction on the immune response, pathogen community, and individual survival in honey bee colonies in North Carolina, U.S.A. We measured the expression of 4 immune genes and relative abundance of 10 pathogens (2 fungi, 1 bacterium, and 7 viruses), using quantitative real-time PCR (qRT-PCR). We also measured survival of worker bees from these same colonies under laboratory conditions, with and without induced oxidative stress. Our results provide previously unavailable insights into the effects of urbanization and beekeeping on the disease ecology of an economically important pollinator.

## Materials and Methods

### Ethics statement

Worker bees from managed colonies were collected with the permission of the individual beekeepers. All feral and managed colonies were located on private property, and permission of property owners was obtained prior to access; no formal permits were required.

### Study sites and insects

**Fig 1:**
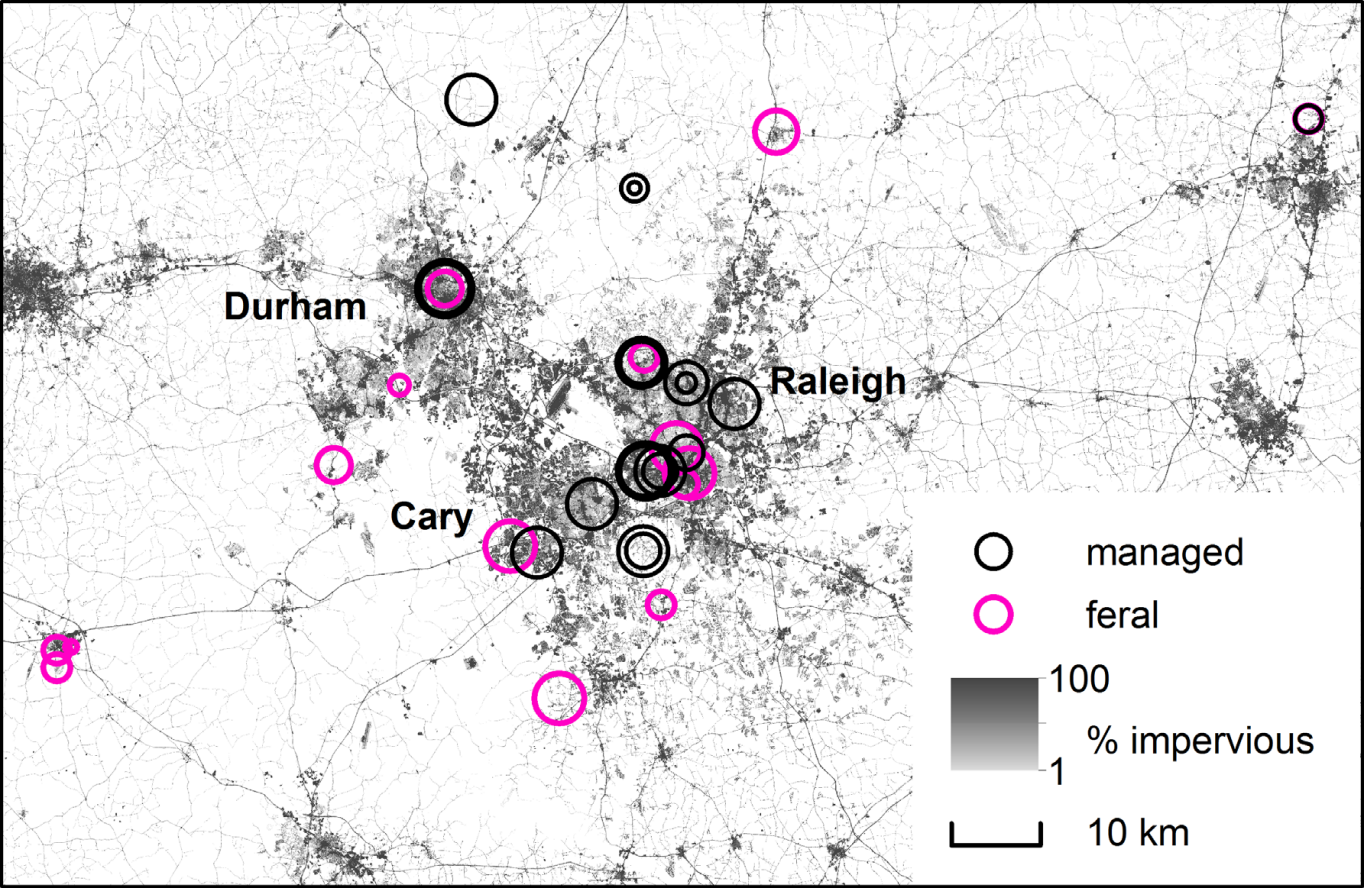
Locations of sampled colonies. Circles represent *N*. *ceranae* relative intensities, with larger circles indicating more intense infections. White areas include no developed impervious surface. (The impervious surface map was generated using the Landsat-derived NLCD 2011 percent developed imperviousness dataset [39]).

Bees were sampled from 24 managed and 15 feral colonies across an urbanization gradient, from the adjacent cities of Raleigh, Durham, and Cary, NC, USA (population ~828,000) to 7 surrounding counties (an area of ~5,250 km^2^). These urban areas are embedded in a complex rural matrix of forest, wetland, grassland, and cropland, where agriculture is a relatively minor land use (S1 Fig). Managed colonies were volunteered by small-scale, non-migratory beekeepers, and locations of feral colonies were provided by beekeepers and the citizen science website www.SaveTheHives.com (Fig 1). Feral colonies lived without human intervention in cavities such as tree holes, and had overwintered in these locations at least once prior to the study. Urbanization was quantified as the proportion of impervious surface within a 1500 m radius of each colony, using the NLCD 2011 percent developed imperviousness dataset (30 m resolution) [39] in ArcMap 10.0. A 1500 m radius represents a typical average foraging distance for *A*. *mellifera*; poor habitat quality within this radius would impose additional stressful, above-average flight distances [40, 41]. At this radius, study sites ranged from 0.1% to 48.2% impervious surface (equivalent to a range of 0.04 to 0.78 in the arcsine-transformed proportions used in analyses). See S1 and S2 Tables for additional radii. Although the absolute amount of unpaved land decreases with urbanization, the composition of undeveloped land cover was similar throughout our urbanization gradient (S1 Fig).

Samples of 50–80 returning foragers were collected using a sweep net near the entrance of each colony between 13 June and 4 September 2013. Feral and managed colonies from across the urbanization gradient were sampled evenly throughout the season (i.e., sample date and “treatment” were independent). Bees were transported in ventilated 50 mL tubes in a cooler, then housed in groups of up to 30 in Plexiglas cages (10 x 10 x 7 cm) and fed 33% sucrose solution ad libitum in a 34°C incubator with 0:24 h L:D photoperiod. Bees were held for 1–24 h before initiating immune and survival assays.

### Immune challenge

An immune response was induced in 10–20 bees per colony. After cold-anaesthetizing bees by briefly chilling them in a -20°C freezer until immobile, a nylon microfilament (0.176 mm diameter, 3 mm length, RIO Products, Powerflex Tippet 4x) was inserted into each bee’s abdomen between tergites 4 and 5. Prior to insertion, probes were roughened with sandpaper, sterilized in ethanol, and dipped in 0.5 mg/mL lipopolysaccharide (Sigma Life Sciences *Escherichia coli* 0128:B12, L2755) in honey bee saline. Probes were removed after 4 h and bees were returned to incubation for ~20 h to allow for immune protein production. Immune gene expression at this endpoint represents response to the probe as well as to pathogens the bees recently experienced in the environment. About 24 hours after probe insertion, bees were transferred to -80°C for storage until RNA extraction.

### Abundance of immune proteins and pathogens

Relative abundance of 4 immune-protein transcripts and 10 pathogens was assayed using qRT-PCR. Target immune genes were abaecin (ABA), defensin-1 (DEF), hymenoptaecin (HYM), and prophenoloxidase (PPO). ABA, DEF, and HYM are antimicrobial peptides induced in response to injury or infection; they function by disrupting microbial cell membranes [42]. PPO underlies melanogenesis, a general immune response that combats viruses, fungi, and other pathogens [36]. The pathogens assayed were the fungi *N*. *apis* and *N*. *ceranae*, the bacterium *P*. *larvae*, and seven viruses: Acute Bee Paralysis Virus, Black Queen Cell Virus, Chronic Bee Paralysis Virus, Deformed Wing Virus, Israeli Acute Paralysis Virus, Kashmir Bee virus, and Sacbrood Virus.

RNA was extracted from colonies in which at least 9 individuals survived for 24 hours after probe insertion (*n* = 15 feral and 20 managed colonies). To extract RNA, abdomens were removed from 9–10 bees per colony, pooled in a clean sandwich bag with 1 mL of TRIzol reagent (Life Technologies), and homogenized with a rolling pin; 250 μL of the resulting mixture were added to a 1.5 mL microcentrifuge tube containing 750 μL TRIzol reagent. The TRIzol manufacturer’s protocol was modified as follows: for phase separation and RNA precipitation, centrifugation was at 16,260 g; for the RNA wash, centrifugation was at 6,532 g; and the RNA pellet was washed twice before suspension in RNAse/DNase-free water and storage at -80°C. RNA quantity and quality were checked using a NanoDrop spectrophotometer (ND-1000 V3.8.1, Thermo Scientific) and cDNA was prepared for each colony using 1.6 μg of RNA template, Superscript II reverse transcriptase (Invitrogen), and random 7-mer primers.

qRT-PCR was performed in 96-well plates using a StepOnePlus Real-Time PCR System (software V2.3, Applied Biosystems). Each reaction contained 5 μL Power SYBR Green Master Mix (Applied Biosystems), 1 μL each of forward and reverse primers (10 μM) (S3 Table), 2 μL RNAse/DNAse-free water and 1 μL cDNA template. All reactions were performed in duplicate with water blanks and subjected to a thermal program of 95°C for 10 min followed by 40 cycles of 94°C for 20 s, 60°C for 30 s, 72°C for 1 min, and 78°C for 20 s. The program ended with 95°C for 15 s, 60°C for 1 min, and 95°C for 15 s. All virus assays were run together as a set with the reference gene, β-actin, while all immune proteins, *Nosema* species and *P*. *larvae* were run in a second set with the same endogenous control. Actin was a suitable reference gene because its C_T_ value did not vary with urbanization or management.

StepOnePlus software was used to calculate the difference in threshold cycle values (ΔC_T_) between each target and β-actin. The ΔC_T_ values were averaged across duplicate reactions after appropriate quality controls for reaction conditions, and relative abundance of each target transcript in each colony was initially calculated as 2^-ΔCT^ (with further normalization described for specific analyses below) [43]. Hereafter we refer to measures of relative abundance of pathogen transcripts as “pathogen intensity.”

### Survival

Laboratory survival in the presence and absence of induced oxidative stress was measured using a separate sample of worker bees from the same colonies. Oxidative stress was induced by injecting the abdomens of cold-anaesthetized bees (17–26 bees per colony) with 2 μL of 6 mg/mL paraquat (Fluka, analytical standard) in honey bee saline using a sterilized microsyringe (Gastight, Hamilton) [44]. This method and dosage followed those reported in another honey-bee study [29]. Another 9–13 bees per colony received 2 μL saline only. Bees’ scuta were marked with colored paint to indicate treatment group (paraquat or control) and bees were incubated in one communal cage per colony. The number of surviving bees in each treatment was recorded every 12 hours for 96 hours after injection.

### Data analysis

Data were tested for effects of urbanization, management, and their interaction on immune protein production, pathogen community composition, and survival. When the interaction term was not significant, it was removed and the marginal (type III) effects of the two main terms examined. In all analyses, the colony was the unit of replication (qRT-PCR data were collected on pooled, colony-level samples), urbanization was measured as the arcsine-transformed proportion impervious surface within 1500 m of each colony, and management was a categorical variable with two levels, feral and managed. Permutation tests used 9999 randomizations of the data except where otherwise indicated.

The 4 immune-protein transcripts were analyzed as a multivariate response using a redundancy analysis (RDA) in the ‘vegan’ package of the R 3.0.0 computing environment for Windows [45, 46]. RDA is a form of multivariate multiple regression that assumes a linear relationship between predictor and response variables, and tests significance with a permutation test [47]. For analysis, target transcript abundance was further normalized to the single rarest detected immune transcript using the 2^-ΔΔCT^ method [43]. Normalizing to the rarest detected transcript generated a tractable multivariate dataset in which all elements were non-negative and measured in the same “units” across transcripts. Data were log (x+1) transformed to meet the assumption of linearity, then centered and scaled to equalize the contribution of all 4 transcripts to the RDA regardless of their overall abundance. The Euclidean distances among colony-level immune responses used in the RDA were tested for spatial autocorrelation using a Mantel test and none was found (*p* = 0.79). Because we were not interested in the potential seasonality of immune response, the RDA was conditioned on the Julian date of sampling; that is, the RDA was a partial RDA after removing any effect of date. Feral and managed samples were further tested for homogeneity of multivariate dispersion using the function betadisper to obtain an analogue of Levene’s test.

The significant effect of management in the RDA was explored using post-hoc univariate tests for the effect of management on each transcript separately. Here, 2^-ΔΔCt^ was recalculated for each transcript using the mean ΔC_T_ for that transcript in managed hives as the calibrator [43]. With this manipulation, results represent fold-change in transcript expression in feral colonies relative to managed colonies. All 4 transcripts individually satisfied *t*-test assumptions and were compared in JMP Pro V10. These tests are presented without Bonferroni correction as an aid to interpreting the RDA.

The same analytical approach was used for pathogens as for immune proteins, with the following modifications. Data were analyzed using distance-based RDA (db-RDA, vegan function capscale) with Jaccard (Ružička) distances, since the Euclidean distances of RDA are misleading when the data include many zeros [47]. Because Jaccard distances do not weight differences in abundant transcripts over those in rare transcripts, data were not scaled. The Jaccard distance matrix was tested for spatial autocorrelation using a Mantel test, which detected a trend toward spatial association among disease communities (*p* = 0.06). Available methods for partitioning multivariate community variance between spatial and environmental components are unreliable [48], so we note the trend but did not attempt to correct for it in the db-RDA.

The db-RDA detected significant effects of urbanization and management on pathogen communities, and these effects were further examined for individual pathogens. Data did not satisfy assumptions of ANOVA, so permutation tests were performed using a randomization wrapper around the GLM procedure of SAS V9.3, with 999 randomizations of the data (SAS Institute, Cary, NC) [49]. We also computed the prevalence, with exact 95% confidence intervals, for each pathogen in feral and managed populations using the R library ‘epiR’[50].

We expected to see links between colonies’ immune response and pathogen infections. Specifically, all 4 immune proteins are expected to interact with fungi, while only PPO is expected to interact with viruses [36, 51]. db-RDAs, again conditioned on date, were performed to test for effects of all 4 immune transcripts on the two fungi, and for effects of PPO on the seven viruses. Because HYM and DEF were collinear (VIF > 5), HYM was excluded as a predictor in our model for fungi.

A Cox proportional hazards model was fit, with management and impervious surface as fixed effects, using the coxph function in the R package ‘survival’ [52]. The nonproportionality test (function cox.zph) detected no violations of the assumption of equal proportional hazards (management: χ^2^ = 0.005, *p* = 0.95; urbanization: χ^2^ = 0.67, *p* = 0.41). The explanatory power of two versions of the model—the additive model with two fixed effects and the model with an interaction term—was compared using a restricted maximum likelihood test.

## Results

### Immunity

**Fig 2:**
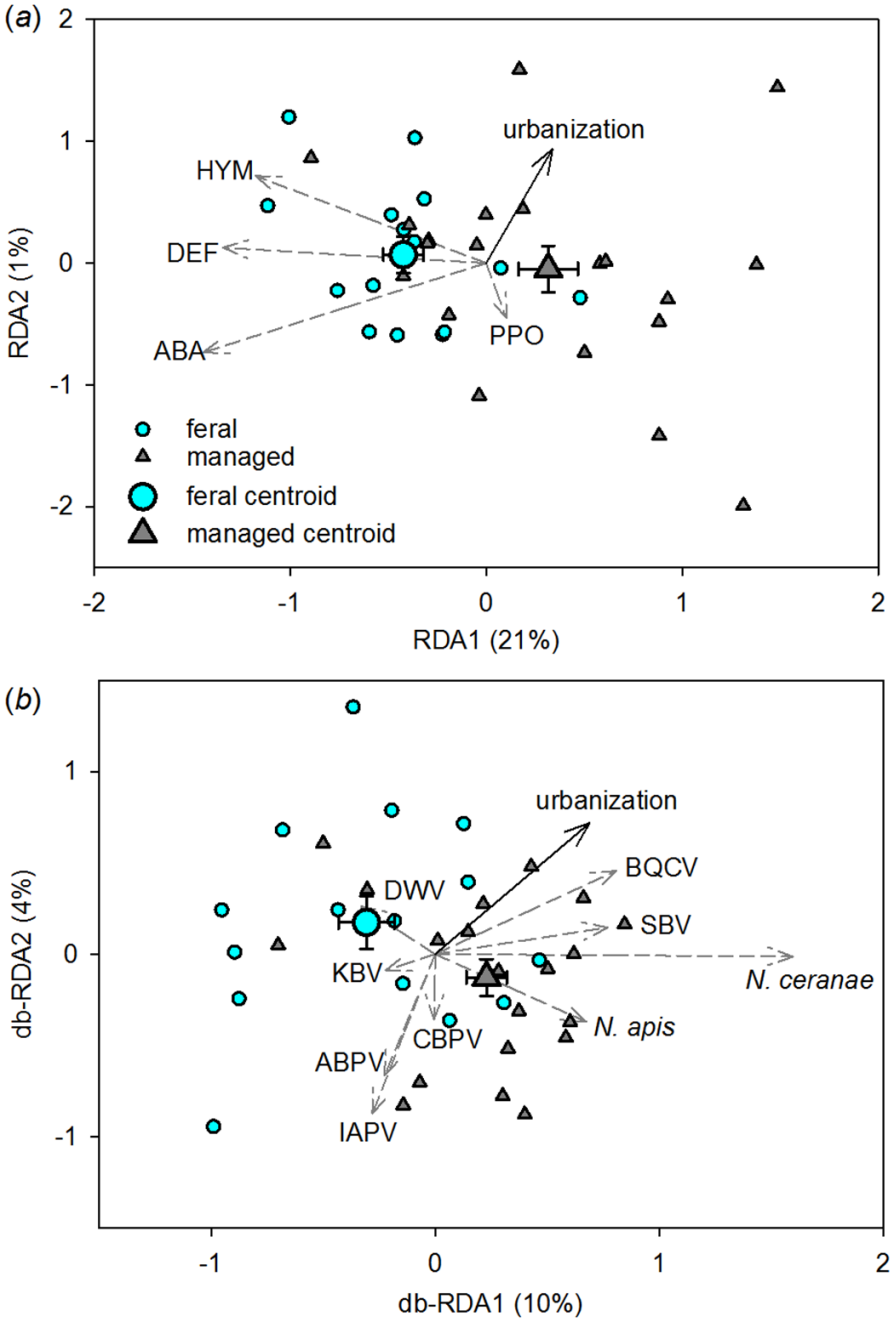
Ordination of immune protein transcripts (A) and disease transcripts (B) in response to urbanization and management. Arrows indicate the direction of increase of continuous variables in multivariate space; solid black arrows are predictors and dashed gray arrows are responses. The wider the angle between a pair of arrows, the less correlated the variables they represent. Each small symbol represents one colony, with distances between symbols approximating dissimilarity in immune transcript composition (A) or disease composition (B) among colonies. Centroids represent the categorical effect of management as the mean responses of feral and managed colonies ± SEM. Immune transcript abbreviations: ABA, abaecin; DEF, defensin; HYM, hymenoptaecin; PPO, prophenoloxidase. Disease abbreviations: ABPV, Acute Bee Paralysis Virus; BQCV, Black Queen Cell Virus; CBPV, Chronic Bee Paralysis Virus; DWV, Deformed Wing Virus; IAPV, Israeli Acute Paralysis Virus; KBV, Kashmir Bee Virus; SBV, Sac Brood Virus.

Overall, feral honey bee colonies expressed immune genes at higher and more consistent levels than did managed bees following an immune challenge (the insertion of a lipopolysaccharide-coated probe). After removing the nonsignificant urbanization x management interaction (*p* = 0.65), the final redundancy analysis (RDA) model accounted for 22% of the total variation in immune protein production (Fig 2, whole model *p <* 0.01) and indicated an effect of management (*p <* 0.01) but not of urbanization within 1500 m of the colonies (*p* = 0.29). This result was consistent when urbanization was measured at different radii around the colonies (S1 Table). Also note that, although the effect of date was removed from this analysis using partial RDA, an alternate version of the model that included date as a main effect detected no significant effect of sampling date on immune gene expression (*p* = 0.28).

Managed colonies were more dispersed around their group centroid than were feral colonies (*F* = 4.95, *p <* 0.05), indicating that variability in immune response was greater among managed colonies. Feral bees transcribed, on average, 1.8 times more hymenoptaecin (*t* = -2.58, *p <* 0.05), 1.9 times more abaecin (*t* = -2.95, *p <* 0.01), and 1.7 times more defensin (*t* = -3.00, *p <* 0.01) than did managed bees, but they did not differ in prophenoloxidase expression (*t* = 0.73, *p* = 0.47) (S2 Fig).

### Disease

The colony-level pathogen assemblage varied with urbanization (*p <* 0.05) and management (*p <* 0.05), but not their interaction (*p* = 0.13; Fig 2, whole model *p <* 0.01). This effect was detected only when urbanization was measured within 1000 m or 1500 m of the colony (S2 Table). Specifically, the intensity of *N*. *ceranae* and Black Queen Cell Virus (BQCV) infections increased with urbanization (Fig 2, Table 1). Responses among the remaining pathogens were weak and variable. Although the effect of date was removed from the analysis using partial db-RDA, an alternate version of the model that included date as a main effect detected no significant effect of sample date on the disease community (*p* = 0.39).

**Table 1:** Results of permutation tests for effects of urbanization and management on the intensity of nine pathogens.

Disease	Whole model	Management		Urbanization	
	*p*	Direction*	*p*	Direction*	*p*
*Nosema apis*	**0.072**	+	**0.040**	+	0.311
*Nosema ceranae*	**0.025**	+	**0.039**	+	**0.037**
ABPV	0.430	+	0.733	–	0.209
BQCV	**0.031**	+	0.187	+	**0.027**
CBPV	0.218	+	0.218	–	0.199
DWV	0.789	–	0.546	–	0.829
IAPV	0.174	+	0.605	–	**0.068**
KBV	0.167	–	0.284	–	0.140
SBV	0.149	+	0.197	+	0.153

*A positive (+) effect indicates that a disease transcript is more abundant in colonies subject to management or to increasing urbanization.

**Fig 3:**
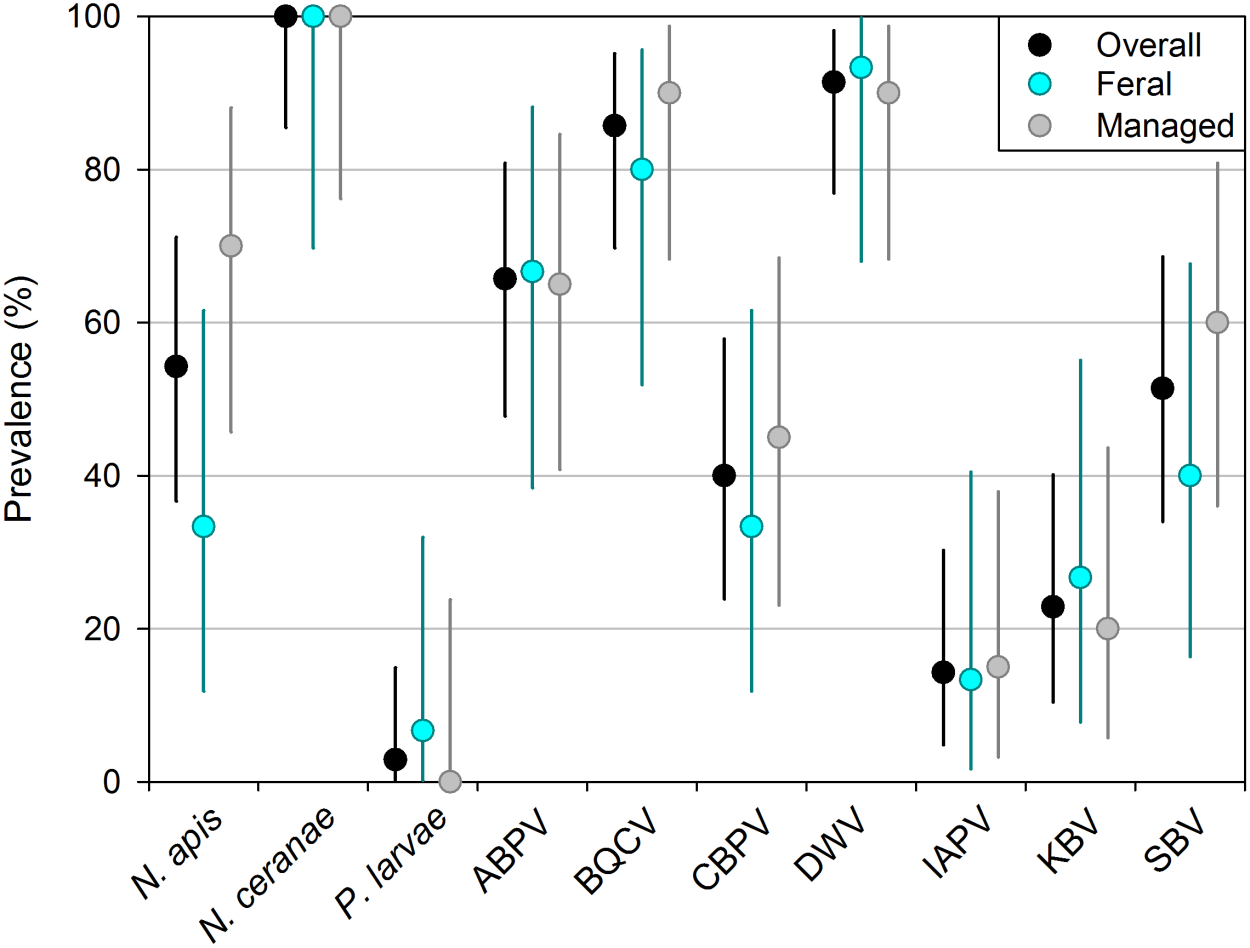
Prevalence of 10 honey bee pathogens in our whole sample and in the feral and managed subsets. Error bars are exact 95% confidence intervals and disease abbreviations are as in Fig 2.

*Nosema apis* and *N*. *ceranae* were more abundant in managed colonies than in feral ones, and 5 of the 7 viruses showed nonsignificant effects in the same direction (Fig 2, Table 1, S3 Fig). We detected a strong inverse relationship between antimicrobial peptide transcription and *N*. *ceranae* intensity (db-RDA whole model *p <* 0.05, S4 Fig) but no relationship between PPO and virus intensity (db-RDA *p* = 0.15). Overall, we detected 10 disease agents, with prevalence ranging from 100% for *N*. *ceranae* to 2.9% for *Paenibacillus larvae*, which appeared in only one (feral) colony (Fig 3). Individual colonies harbored 2 to 8 disease agents (feral mean 4.93, managed mean 5.55, *t* = 1.18, *p* = 0.25).

### Survival

**Fig 4:**
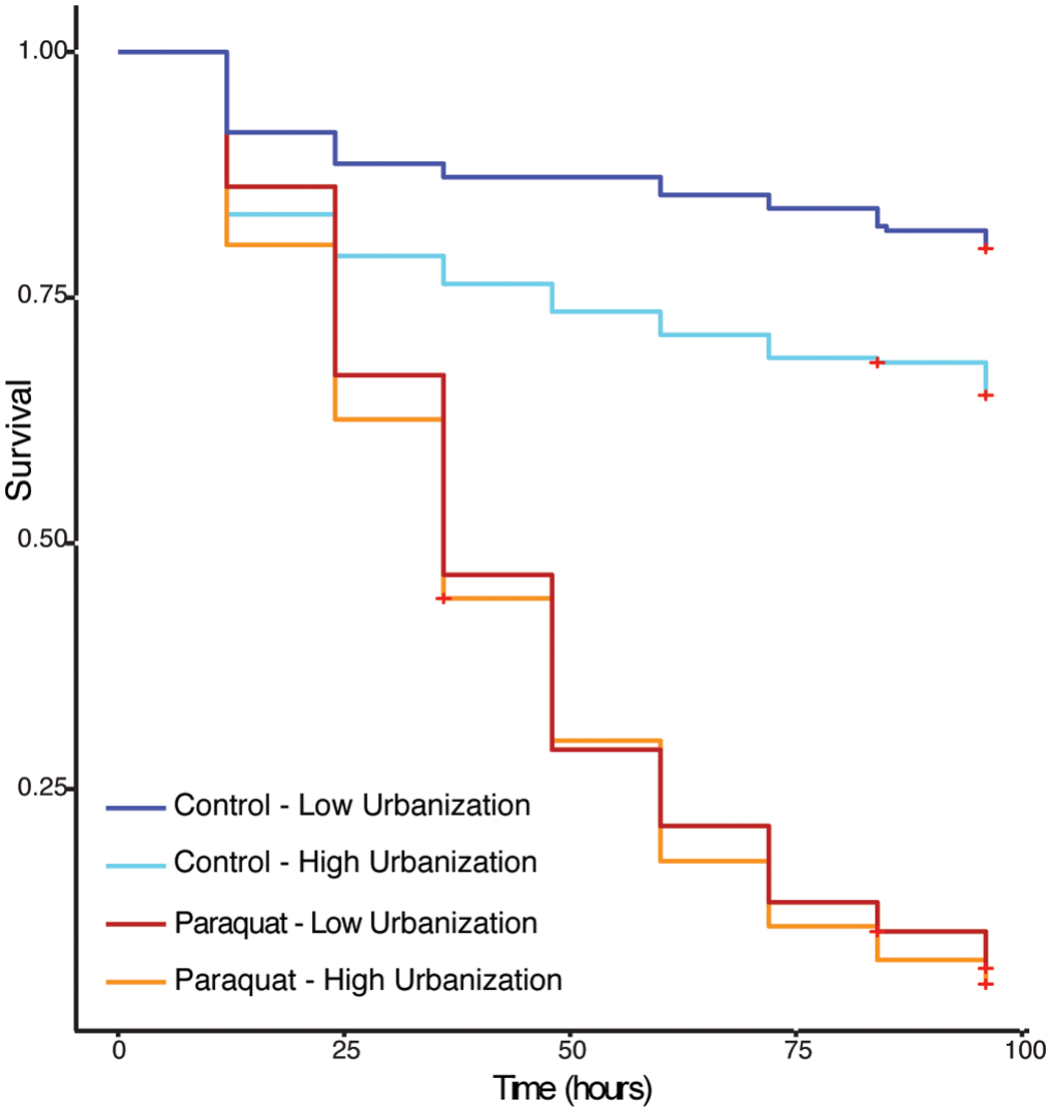
Survival plot of control and paraquat treated bees. Red crosses indicate censored individuals. (Cox model for control bees: *p <* 0.01; Cox model for paraquat bees: *p <* 0.05). Although urbanization was analyzed as a continuous variable (percent impervious surface) in the Cox model, it is represented here as two categories for visual clarity; the categories represent sites above and below the median level of urbanization.

Injection with the oxidative stress agent paraquat greatly reduced honey bee survival over our 96-hour assay (*p <* 0.001, S4 Table), but with a paraquat x urbanization interaction (*p <* 0.05). We therefore examined the effects of urbanization in control bees and paraquat-injected bees separately (Fig 4, Table 2, S5 Table). Among control bees, the probability of survival decreased 4-fold per unit of urbanization, where urbanization was quantified as arcsine-transformed proportion of impervious surface. This effect represents a 3-fold decline in survival across our sampled gradient. Although all paraquat-injected bees died rapidly, the cost of urbanization was still detectable with a 1.5-fold decrease in probability of survival per unit urbanization (Table 2).

**Table 2:** Results of Cox regression model for additive fixed effects of urbanization and management on survival.

Treatment	Predictor	b*	se	*p*-value	e^b^ †	95% CI around e^b^
Control	Management	0.109	0.192	0.569	1.115	(0.766, 1.624)
	Urbanization	1.409	0.491	0.004	4.093	(1.562, 10.722)
Paraquat	Management	0.021	0.073	0.769	1.021	(0.886, 1.177)
	Urbanization	0.407	0.176	0.021	1.502	(1.063, 2.123)

*b is the regression coefficient of each variable.

^†^e^b^ is interpreted as the multiplicative effect of the hazard (e^b^ = 1 indicates no hazard; e^b^ < 1 indicates decreased hazard; e^b^> 1 indicates increased hazard)

## Discussion

Our results show that urbanization alters the disease ecology of feral and managed honey bees by increasing pathogen loads, particularly favoring BQCV and the fungal parasite *N*. *ceranae*. This effect does not appear to be mediated by immunity, as measured by immune-gene expression, which was independent of urbanization. Coping with urban habitats could impose tradeoffs on immune mechanisms not measured here, such as RNAi, but urbanization may also promote disease independently of immunity by enhancing disease transmission, virulence, or persistence. For example, in temperate climates, urban warming could improve viability of *N*. *ceranae* spores, which suffer reduced germination after exposure to temperatures of 4°C or colder [32, 53]. This sensitivity is thought to shape the geographic distribution of *N*. *ceranae* on a continental scale [13, 32], and it may do so on a local scale as well. Urban habitats are typically 1–3°C warmer than their rural surroundings (e.g., [54]), potentially reducing exposure of *N*. *ceranae* spores to damaging winter cold. *N*. *apis* lacks this cold sensitivity, consistent with its non-response to urbanization [32, 53]. Urbanization may also enhance pathogen transmission by altering interactions among bees. Resource patchiness or high population densities force bees from different colonies and species to interact more frequently with one another and with shared resources [27, 55–57]. Diseases may be vectored by these shared resources, as has been shown for viruses (including BQCV) on flowers [55]. There was a trend toward spatial correlation among the disease communities we measured, pointing to the need for studies that assess the role of contagion in the urban distribution of bee pathogens.

Our results also show that survival of feral and managed worker bees declined with urbanization. This decline was more pronounced in control (saline-injected) bees than in paraquat-injected bees (represented by a paraquat x urbanization interaction; Fig 4, S4 Table). However, we refrain from interpreting this interaction in terms of oxidative stress, because all paraquat-injected bees died rapidly, suggesting that they died of direct toxicity rather than accumulated oxidative stress. Although our dosage followed published methods [29], a lower dose may have been appropriate for our populations. For these reasons, we exclude these bees from further discussion; instead we call attention to the saline-injected control bees, whose probability of survival declined 3-fold across our urbanization gradient. The specific causes of this striking effect remain to be tested, and future work should consider not only oxidative stress but also disease, since colony-level disease intensity (particularly for *N*. *ceranae* and BQCV) increased with urbanization while survival declined. (This association was not tested directly here, since survival and disease intensity were measured in separate samples of worker bees.)

We measured urbanization as the proportion of impervious surface within a 1500 m radius of each colony, based on the average foraging flight distance of honey bee workers. Although a worker bee may fly up to 10 km, areas within 1–2 km of the colony are the most heavily foraged [41], and landscape composition at this scale is likely to shape the majority of worker bees’ interactions with the environment and with other bees [40, 41]. Analysis of urbanization within different radii (100–3000 m) indicated that our a priori choice of 1500 m was reasonable. The effect of urbanization on disease was detectable only at 1000 m and 1500 m (S2 Table), while urbanization did not affect immunity at any radius (S1 Table).

The consequences of urbanization and management were additive, not interactive; that is, urbanization imposed similar changes on feral and managed colonies in terms of their pathogen communities and worker survival. Management itself also dramatically altered pathogen intensity and immune-gene expression. Both *Nosema* parasites were more abundant in managed colonies, and 5 of 7 viruses trended in the same direction. Unlike urbanization, management was associated with weakened and variable immune responses and, by extension, greater susceptibility of honey bee workers to disease. Whether these differences in immune response are genetic or environmental remains to be seen; some isolated feral populations in the U.S. are genetically distinct from managed bees [23, 58, 59], but effects of colony housing alone can also alter honey bee immune responses [60].

We found a strong inverse relationship between antimicrobial peptide transcription and *N*. *ceranae* intensity. Although insect antimicrobial peptides are effective against fungi and are typically up-regulated in response to fungal infection [61], feedbacks between immunity and disease are complex. Several studies have found transient immunosuppressive effects of *N*. *ceranae*, such that the relatively weak immune response in our managed bees could be a consequence, rather than a cause, of high-intensity *N*. *ceranae* infections [62–64]. On the other hand, a study of an *N*. *ceranae*-tolerant honey bee strain in Denmark showed that both resistant and tolerant bees up-regulated immune pathways in the first 5 days after infection, after which only tolerant bees had a strong immune response [65]. This study supports an interpretation in which feral bees, with higher expression of immune genes, better suppressed *N*. *ceranae*. Future studies should further examine cause and effect in the patterns we observed to inform management practices that will reduce severity of *N*. *ceranae* infections.

Our results differ from those of an Australian study, which identified weaker immune response in feral than managed bees [60]. We suspect that this difference arises from vastly different selective pressures on the bees in the two studies, because neither *Varroa* mites nor *N*. *ceranae* was present in the Australian populations [66]. Our pathogen results are consistent with studies that identified lower intensity or prevalence of *Nosema* parasites in feral bees than managed bees in the U.S. and Australia [22, 67, 68], and a similar trend in Europe [21]. We detected an extraordinarily high prevalence of *N*. *ceranae* (100% of colonies infected) relative to other studies using similar, PCR-based methods in the continental U.S., most of which have reported 50–70% prevalence [20, 69], or 88% in Hawaii [70]. These differences may reflect continued establishment of the parasite or a high density of honey bee colonies in our study area. We are aware of only one other study examining viral loads in feral bees; Thompson et al. [21] identified higher levels of DWV in feral bees relative to managed bees in the UK. We identified a weak, nonsignificant effect in the same direction. Since both studies relied on relative quantification, this difference could signify that feral bees in our study are less burdened with DWV, managed bees more so, or both. Distinguishing between these alternatives could suggest best management practices among beekeepers.

Animal pollinators, particularly managed honey bees, provide more than $170 billion in pollination services to global agriculture each year [71]. This service is threatened by environmental factors including emerging infectious diseases. We found that urbanization and management worsen honey bee pathogen loads through additive effects. More intense infections in managed colonies are linked with weaker immune responses. Certain populations of feral bees, although initially devastated by emerging diseases, are now coping with these threats. Continued study of the genetic and environmental underpinnings of their strong immune response may illuminate a way forward for honey bee conservation.

Urbanization also increased pathogen intensity, but did not affect immunity, and we suggest that urban environments instead favor pathogen transmission. In cities, contact among human, domestic, and wild animals facilitates cross-species spillover of vertebrate pathogens [3, 5]. Urbanization may present similar threats to pollinators. Given the high pathogen burden of urban, managed bees, and the density of bees on shared, limited floral resources [9, 27, 56], cities could present ideal conditions for pathogen spillover among honey bees and wild pollinators.

## Supporting Information

S1 Data Sample dates, impervious surface, and qPCR data. (XLSX)

S2 Data Spatial distances among colonies (km). (XLSX)

S3 Data Survival data. (XLSX)

S1 Fig Undeveloped land cover in the study area. (DOCX)

S2 Fig Relative abundance of immune protein transcripts in feral and managed colonies. (DOCX)

S3 Fig Relative abundance of individual pathogens with respect to urbanization and management. (DOCX)

S4 Fig db-RDA ordination of immune protein transcripts and *Nosema* infection intensity, illustrating the strong inverse relationship between *N*. *ceranae* and defensin. (DOCX)

S1 Table Summary of immune RDA results, repeated using urbanization at different radii. (DOCX)

S2 Table Summary of pathogen db-RDA results, repeated using urbanization at different radii. (DOCX)

S3 Table Primer sequences. (DOCX)

S4 Table Full Cox model including all parameters and interactions. (DOCX)

S5 Table Separate Cox models for paraquat and control groups, including interaction terms. (DOCX)
