# Supplementary material for: Urbanization Increases Pathogen Pressure on Feral and Managed Honey Bees
Source: PLoS One. 2015 Nov 4;10(11):e0142031. doi: 10.1371/journal.pone.0142031 (PMC4633120; doi:10.1371/journal.pone.0142031)
Supplement: S1 Fig — (DOCX) [file pone.0142031.s004.docx]

**S1 Fig. Undeveloped land cover in the study area.** Mean composition of undeveloped land^a^ within a 1500 m radius of (A) all 39 colonies, (B) the 10 most urban colonies, and (C) the 10 most rural colonies in the study; note that developed cover is represented by the percent imperviousness measure used throughout the main text and is not included here.


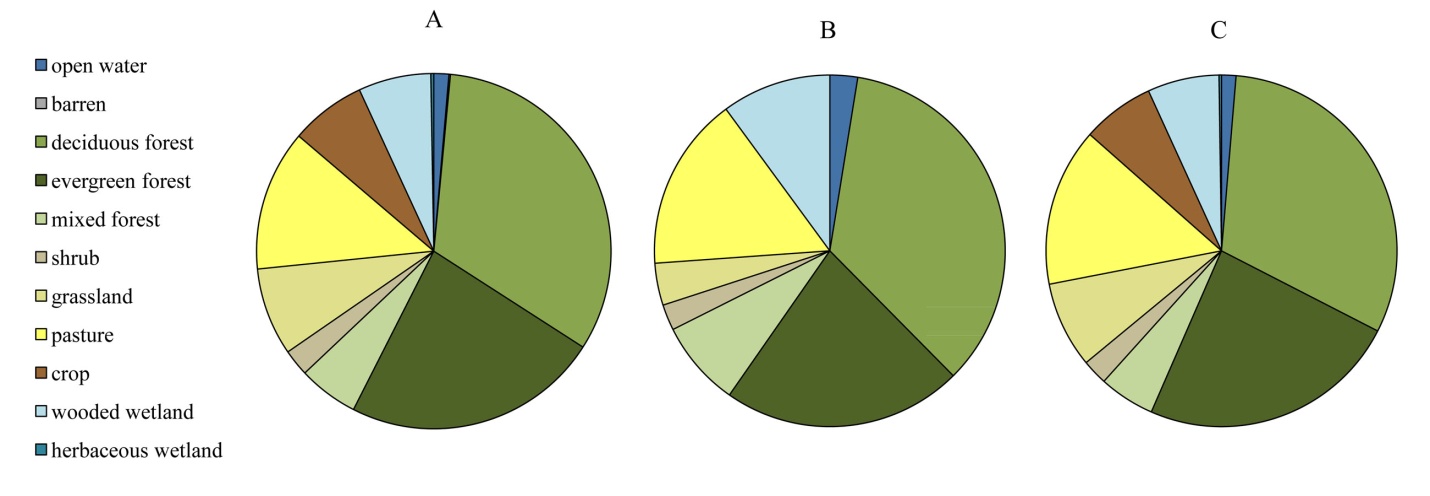


^a^ Data extracted from the 2011 National Land Cover Database [1] using ArcMap 10.0.

1. Jin S, Yang L, Danielson P, Homer C, Fry J, Xian G. A comprehensive change detection method for updating the National Land Cover Database to circa 2011. Remote Sens Environ. 2013;132:159-175.
