# Supplementary material for: Urbanization Increases Pathogen Pressure on Feral and Managed Honey Bees
Source: PLoS One. 2015 Nov 4;10(11):e0142031. doi: 10.1371/journal.pone.0142031 (PMC4633120; doi:10.1371/journal.pone.0142031)
Supplement: S2 Fig — (DOCX) [file pone.0142031.s005.docx]

**S2 Fig. Relative abundance of immune protein transcripts in feral and managed colonies.** As described in the main text, each transcript was normalized separately using the mean ΔCT for that transcript in managed hives as the calibrator. (Peptide abbreviations are as in Fig. 2 in the main text.)
