# Supplementary material for: Urbanization Increases Pathogen Pressure on Feral and Managed Honey Bees
Source: PLoS One. 2015 Nov 4;10(11):e0142031. doi: 10.1371/journal.pone.0142031 (PMC4633120; doi:10.1371/journal.pone.0142031)
Supplement: S3 Fig — (DOCX) [file pone.0142031.s006.docx]

**S3 Fig. Relative abundance of individual pathogens with respect to urbanization and management.** Blue symbols represent feral colonies; gray symbols represent managed colonies. Relative abundance was normalized to the overall rarest detected disease transcript for multivariate analysis (main text) and the same scaling is retained here. Trendlines are included for pathogens that were highlighted as most important in post-hoc univariate randomization tests (Table 1 in the main text).
