# Supplementary material for: Urbanization Increases Pathogen Pressure on Feral and Managed Honey Bees
Source: PLoS One. 2015 Nov 4;10(11):e0142031. doi: 10.1371/journal.pone.0142031 (PMC4633120; doi:10.1371/journal.pone.0142031)
Supplement: S4 Fig — (DOCX) [file pone.0142031.s007.docx]

**S4 Fig. db-RDA ordination of immune protein transcripts and *Nosema* infection intensity, illustrating the strong inverse relationship between *N. ceranae* and defensin.** Each symbol represents one colony, and arrows indicate the direction of increase of continuous variables in multivariate space; the smaller the angle between two arrows, the more positively correlated the variables they represent. *N. apis* is essentially uncorrelated with other variables, such that its arrow is invisibly short on this plot. (Peptide abbreviations are as in Fig. 2 in the main text.)
