# Supplementary material for: Urbanization Increases Pathogen Pressure on Feral and Managed Honey Bees
Source: PLoS One. 2015 Nov 4;10(11):e0142031. doi: 10.1371/journal.pone.0142031 (PMC4633120; doi:10.1371/journal.pone.0142031)
Supplement: S1 Table — (DOCX) [file pone.0142031.s008.docx]

**S1 Table. Summary of immune RDA results, repeated using urbanization at different radii.** Regardless of the radius used, results are qualitatively similar: Immune gene expression declines with management but is unaffected by urbanization or the interaction.

|  | Radius (m) | | | | |
| --- | --- | --- | --- | --- | --- |
|  | 100 | 1000 | 1500 | 2000 | 3000 |
| Whole model *p* | < 0.05 | < 0.05 | < 0.05 | < 0.05 | < 0.05 |
| Interaction *p* | 0.83 | 0.64 | 0.65 | 0.66 | 0.74 |
| Whole model *p* without interaction | < 0.01 | < 0.01 | < 0.01 | < 0.01 | < 0.01 |
| Urbanization *p* | 0.43 | 0.42 | 0.29 | 0.24 | 0.25 |
| Management *p* | < 0.01 | < 0.01 | < 0.01 | < 0.01 | < 0.01 |
| % Explained | 20.9 | 20.9 | 21.7 | 22.2 | 22.1 |
| Range in % imperv across sites | 0 - 71.2 | 0.2 - 61.4 | 0.1 - 48.2 | 0.4 - 39.5 | 0.4 - 32 |
