# Supplementary material for: Urbanization Increases Pathogen Pressure on Feral and Managed Honey Bees
Source: PLoS One. 2015 Nov 4;10(11):e0142031. doi: 10.1371/journal.pone.0142031 (PMC4633120; doi:10.1371/journal.pone.0142031)
Supplement: S2 Table — (DOCX) [file pone.0142031.s009.docx]

**S2 Table. Summary of pathogen db-RDA results, repeated using urbanization at different radii.** The effect of management remains regardless of the urbanization radius; the effect of urbanization is detected only at intermediate radii.

|  | Radius (m) | | | | |
| --- | --- | --- | --- | --- | --- |
|  | 100 | 1000 | 1500 | 2000 | 3000 |
| Whole model *p* | < 0.05 | < 0.01 | < 0.01 | < 0.01 | < 0.05 |
| Interaction *p* | 0.16 | 0.21 | 0.13 | 0.11 | 0.11 |
| Whole model *p* (without interaction) | < 0.05 | < 0.01 | < 0.01 | < 0.05 | < 0.05 |
| Urbanization *p* | 0.15 | < 0.05 | < 0.05 | 0.08 | 0.11 |
| Management *p* | < 0.05 | < 0.05 | < 0.05 | < 0.05 | 0.05 |
| % Explained | 10.2 | 12.6 | 11.3 | 10.8 | 10.5 |
