# Supplementary material for: Urbanization Increases Pathogen Pressure on Feral and Managed Honey Bees
Source: PLoS One. 2015 Nov 4;10(11):e0142031. doi: 10.1371/journal.pone.0142031 (PMC4633120; doi:10.1371/journal.pone.0142031)
Supplement: S3 Table — (DOCX) [file pone.0142031.s010.docx]

**S3 Table. Primer sequences.**

| Target | Primer sequence | | Reference | |
| --- | --- | --- | --- | --- |
|  | Forward | Reverse |  | |
| Abaecin | CAGCATTCGCATACGTACCA | GACCAGGAAACGTTGGAAAC | | 1 |
| Hymenoptaecin | CTCTTCTGTGCCGTGCATA | GCGTCTCCTGTCATTCCATT | | 1 |
| Defensin-1 | TGCGCTGCTAACTGTCTCAG | AATGGCACTTAACCGAAACG | | 1 |
| Prophenoloxidase | AGATGGCATGCATTTGTTGA | TTGCGTTGTTGATTGGTTGT | | 1 |
| ABPV | GCCCAGACAAGCGCAGTACT | AGCACGGAAAACGCGTCTT | | 2* |
| BQCV | CCAGGTTTGTTTGCCGACTTAC | CGTACGGGCCTCGGATAA | | 3* |
| CBPV | CGTTGGTGTTAGCAGGTTTGG | TCACCTGAGCAACGAGTACCAT | | 4* |
| DWV | TTAATCAGCGCTTAGTGGAGGAA | CGCACCTACGCGATGTAAATC | | 3* |
| IAPV | TGCGGCGTTCCGAAATA | TCACTAGTGGACGAAGCGTAGTTC | | 5* |
| KBV | GCATCGAGCGCATTCCA | TACACCTCCTACAATATCAGCAAACC | | 3* |
| SBV | ACCGTTATACGCGTGCTGAAT | TGGCCCCCCGGATATT | | 3* |
| *Paenibacillus larvae* | CGGGAGACGCCAGGTTAG | TTCTTCCTTGGCAACAGAGC | | 6 |
| *Nosema apis* | GCCCTCCATAATAAGAGTGTCCAC | ATCTCTCATCCCAAGAGCATTGC | | 7 |
| *Nosema ceranae* | AAGAGTGAGACCTATCAGCTAGTTG | CCGTCTCTCAGGCTCCTTCTC | | 7 |
| β-actin | CGTTGTCCCGAGGCTCTTT | TGAATACCGCAAGCTTCCATT | | 8* |
|  |  |  |  | |
| *Modified from original report to optimize reaction conditions on ABI 7900 | | |  | |

**References for S4**

1. Evans JD (2006) Beepath: an ordered quantitative-PCR array for exploring honey bee immunity and disease. J Invertebr Pathol 93:135-139.

2. Grabensteiner E, Bakonyi Ts, Ritter W, Pechhacker H, & Nowotny N (2007) Development of a multiplex RT-PCR for the simultaneous detection of three viruses of the honeybee (Apis mellifera L.): acute bee paralysis virus, Black queen cell virus and Sacbrood virus. J Invertebr Pathol 94:222-225.

3. Chen Y, Zhao Y, Hammond J, Hsu H-t, Evans J, & Feldlaufer M (2004) Multiple virus infections in the honey bee and genome divergence of honey bee viruses. J Invertebr Pathol 87:84-93.

4. Ribière M, Triboulot C, Mathieu L, Aurières Cm, Faucon J-P, & Pépin M (2002) Molecular diagnosis of chronic bee paralysis virus infection. Apidologie 33:339-352.

5. Cox-Foster DL, Conlan S, Holmes EC, Palacios G, Evans JD, Moran NA, Quan P-L, Briese T, Hornig M, & Geiser DM (2007) A metagenomic survey of microbes in honey bee colony collapse disorder. Science 318:283-287.

6. Martínez J, Simon V, Gonzalez B, & Conget P (2010) A real-time PCR-based strategy for the detection of Paenibacillus larvae vegetative cells and spores to improve the diagnosis and the screening of American foulbrood. Lett Appl Microbiol 50:603-610.

7. Bourgeois AL, Rinderer TE, Beaman LD, & Danka RG (2010) Genetic detection and quantification of Nosema apis and N. ceranae in the honey bee. J Invertebr Pathol 103:53-58.

8. Chen Y, Higgins J & Feldlaufer M (2005) Quantitative real-time reverse transcription-PCR analysis of deformed wing virus infection in the honeybee (Apis mellifera L.). Appl Environ Microbiol 71:436-441.
