# Supplementary material for: Urbanization Increases Pathogen Pressure on Feral and Managed Honey Bees
Source: PLoS One. 2015 Nov 4;10(11):e0142031. doi: 10.1371/journal.pone.0142031 (PMC4633120; doi:10.1371/journal.pone.0142031)
Supplement: S4 Table — (DOCX) [file pone.0142031.s011.docx]

**S4 Table. Full Cox model including all parameters and interactions.**

| Predictor | b^*^ | se^†^ | *p* | e^b^ǂ | 95% CI |
| --- | --- | --- | --- | --- | --- |
| Treatment (paraquat or control) | 2.904 | 0.424 | < 0.001 | 18.240 | (7.943, 41.887) |
| Management | 0.584 | 0.488 | 0.232 | 1.793 | (0.689, 4.670) |
| Urbanization | 2.218 | 0.830 | 0.008 | 9.187 | (1.806, 46.670) |
| Treatment*Management | -0.597 | 0.517 | 0.249 | 0.551 | (0.200, 1.518) |
| Treatment*Urbanization | -1.904 | 0.895 | 0.033 | 0.149 | (0.026, 0.862) |
| Management*Urbanization | -1.068 | 1.016 | 0.293 | 0.344 | (0.047, 2.516) |
| Treatment*Management*Urbanization | 1.155 | 1.090 | 0.289 | 3.173 | (0.375, 26.849) |

*regression coefficient of each variable

^†^standard error of regression coefficient

ǂhazard ratio (e^b^ = 1 indicates no hazard; e^b^ < 1 indicates decreased hazard; e^b^> 1 indicates increased hazard)
