# Supplementary material for: Urbanization Increases Pathogen Pressure on Feral and Managed Honey Bees
Source: PLoS One. 2015 Nov 4;10(11):e0142031. doi: 10.1371/journal.pone.0142031 (PMC4633120; doi:10.1371/journal.pone.0142031)
Supplement: S5 Table — (DOCX) [file pone.0142031.s012.docx]

**S5 Table. Separate Cox models for paraquat and control groups, including interaction terms.**

| Treatment | Predictor | b* | SE^†^ | *P* | e^b^ǂ | 95% CI |
| --- | --- | --- | --- | --- | --- | --- |
| Control | Management | 0.519 | 0.486 | 0.286 | 1.680 | (0.647, 4.357) |
|  | Urbanization | 2.024 | 0.822 | 0.014 | 7.569 | (1.513, 37.871) |
|  | Management*Urbanization | -0.936 | 1.011 | 0.355 | 0.392 | (0.054, 2.844) |
| Paraquat | Management | -0.013 | 0.171 | 0.942 | 0.988 | (0.706, 1.382) |
|  | Urbanization | 0.344 | 0.337 | 0.307 | 1.411 | (0.728, 2.734) |
|  | Management*Urbanization | 0.086 | 0.396 | 0.828 | 1.090 | (0.502, 2.368) |

*regression coefficient of each variable

^†^standard error of regression coefficient

ǂhazard ratio (e^b^ = 1 indicates no hazard; e^b^ < 1 indicates decreased hazard; e^b^> 1 indicates increased hazard)
